# Identifying *Cis*-Regulatory Sequences by Word Profile Similarity

**DOI:** 10.1371/journal.pone.0006901

**Published:** 2009-09-04

**Authors:** Garmay Leung, Michael B. Eisen

**Affiliations:** 1 University of California Berkeley and University of California San Francisco Joint Graduate Group in Bioengineering, University of California, Berkeley, California, United States of America; 2 Department of Molecular and Cell Biology, University of California, Berkeley, California, United States of America; 3 Howard Hughes Medical Institute, University of California, Berkeley, California, United States of America; University of Toronto, Canada

## Abstract

**Background:**

Recognizing regulatory sequences in genomes is a continuing challenge, despite a wealth of available genomic data and a growing number of experimentally validated examples.

**Methodology/Principal Findings:**

We discuss here a simple approach to search for regulatory sequences based on the compositional similarity of genomic regions and known *cis*-regulatory sequences. This method, which is not limited to searching for predefined motifs, recovers sequences known to be under similar regulatory control. The words shared by the recovered sequences often correspond to known binding sites. Furthermore, we show that although local word profile clustering is predictive for the regulatory sequences involved in blastoderm segmentation, local dissimilarity is a more universal feature of known regulatory sequences in *Drosophila*.

**Conclusions/Significance:**

Our method leverages sequence motifs within a known regulatory sequence to identify co-regulated sequences without explicitly defining binding sites. We also show that regulatory sequences can be distinguished from surrounding sequences by local sequence dissimilarity, a novel feature in identifying regulatory sequences across a genome. Source code for WPH-finder is available for download at http://rana.lbl.gov/downloads/wph.tar.gz.

## Introduction

Rates of transcription from different promoters in animal genomes are influenced by the binding of sequence-specific DNA-binding transcription factors to cognate binding sites within compact regulatory sequences known collectively as *cis*-regulatory modules [1]. However, the identification of CRMs is confounded by our incomplete understanding of the rules that govern the relationship between the organization and composition of regulatory sequences and their function.

Where the transcription factors involved in regulating a battery of genes are known and their binding specificities characterized, regulatory sequences responding to these factors can often be identified [2]–[4], especially when comparative sequence data is used [5]–[6]. However, except in a handful of well-characterized regulatory systems, the binding profiles of the relevant transcription factors are unknown. Furthermore, these methods are most effective where the local concentrations of transcription factor binding sites (TFBSs) in regulatory sequences are high, and such “binding site clustering” is not a universal feature of CRMs [7].

To circumvent this limitation, several methods have been developed to identify shared sequence features of known CRMs and exploit these signals to identify novel instances. The fluffy-tail test takes advantage of a characteristic word distribution of CRMs to identify regulatory sequences [8]. HexDiff uses the hexamer frequencies of co-regulated and of non-regulatory sequences, and has proven to be successful given appropriate positive and negative training sets [9]. LWF groups together words that have similar local word frequencies, building a statistical likelihood profile based on known CRMs that allows for prediction of similar CRMs [10]. Another class of *ab initio* CRM discovery programs looks for CRMs within a set of sequences by stochastically searching for subsequences that are maximally similar, which shows promise in identifying CRMs when a set of co-expressed genes is known (CSam, D2Z-set [11]).

Although the use of auxiliary information can be extremely valuable in predicting CRMs, such information is not always available. We developed a method called WPH-finder, a means to identify co-regulated sequences in the absence of explicit TFBSs, alignments, or large training sets of co-expressed sequences or genes. Given a known *cis*-regulatory module, WPH-finder uses its word composition to search for other putative CRMs with similar word composition. We also show that although stripe CRMs can be recovered by identifying clustered word profiles, neighboring dissimilar word profiles are a more common feature of regulatory sequences.

## Results

### Word profiles of known regulatory sequences recover co-regulated sequences

**Figure 1:**
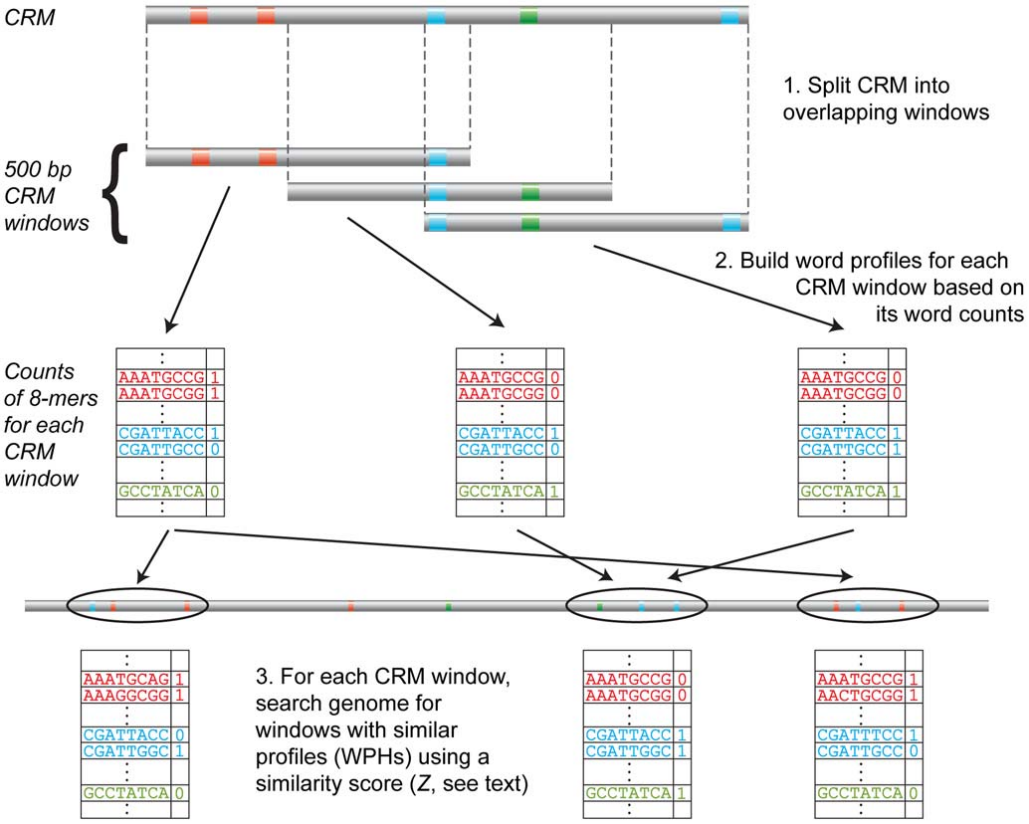
WPH-finder: Finding putative co-regulated CRMs (WPHs). To identify putative co-regulated sequences given a known CRM, we first split the CRM into overlapping windows to allow us to leverage closely linked word or motif combinations. Each of these windows is represented by its word counts, or its word profile, which is then used to identify similar word profiles in the genome. A set of WPHs for a given CRM window consists of genome sequence windows whose word profiles are similar to the word profile of the CRM window, as measured by our similarity score *Z*.

Given a known CRM, we would like to identify similar sequences in the genome as putative co-regulated sequences. To this end, we defined a score (*Z*) that measures the pairwise similarity between two sequences based on their word content. The score measures how likely the words found in one sequence would be found in a second independently-generated sequence. A given sequence is represented by its word profile, or its 8-mer composition, and is associated with a set of genome sequences that have similar word contents, which we refer to as word profile hits (WPHs). A known CRM can thus be used to scan the genome for putative co-regulated CRMs using our WPH-finder program (Figure 1).

To determine whether WPH-finder can accurately detect co-regulated sequences, for a given CRM, we evaluated the degree to which its known co-regulated sequences are overrepresented in its set of WPHs. Our first dataset consists of the stripe CRMs regulating the primary pair rule genes (*eve*, *h*, and *run*) in *Drosophila melanogaster* (Supporting File S1). These well-characterized CRMs are known to share common TFBSs and are all involved in anterior-posterior patterning during embryonic development. The availability of chIP-chip binding data for known regulators of these stripe CRMs [12] allows us to evaluate the predictive power of stripe WPHs on additional test sets, specifically the regions bound by transcription factors BCD, GT, HB, and KR near pair-rule genes. Pair rule genes each exhibit different segmental phasing in response to the concentrations and combinations of maternal (i.e. BCD and HB) and gap (i.e. GT and KR) transcription factors [13], and these bound regions likely share TFBS combinations with the stripe CRMs. Since it is unlikely that the boundaries of experimentally verified CRMs are perfectly annotated, for the purposes of this validation step, we collected WPHs for each 500 bp sequence window (shifted by 100 bp) across 15 kb regions that span known stripe CRMs (see *Methods*). This windowing allows closely linked binding sites to be considered simultaneously. Each sequence window is associated with a set of similar sequences from the genome (WPHs). We assessed the predictive power of a given set of WPHs by determining the significance of its overlap with a set of known regulatory regions.

**Figure 2:**
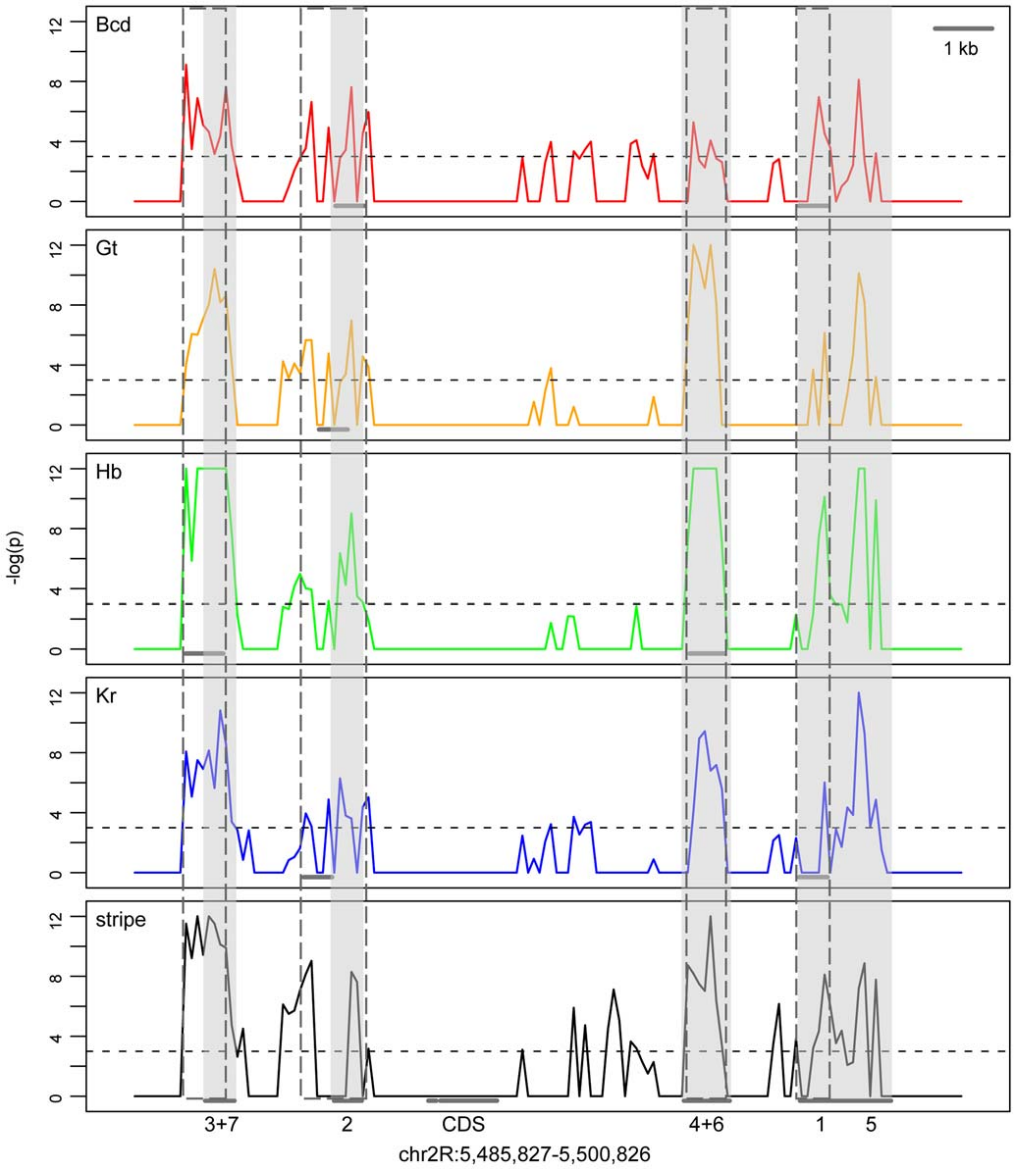
Significance of overlap between eve WPHs and test sets. Each sequence window across the *eve* locus is associated with a set of WPHs. We observe significant overlap between WPHs corresponding to annotated CRMs and our test sets (stripe CRMs and four sets of chIP-chip peaks). Stripe CRMs are shaded in gray, and chIP-chip bounds regions are boxed in a dotted line. For *p*<1e-5, the *p*-value is reported as 6.1e-6 (−log(*p*) = 12). The dashed line represents *p* = 0.05.

**Figure 3:**
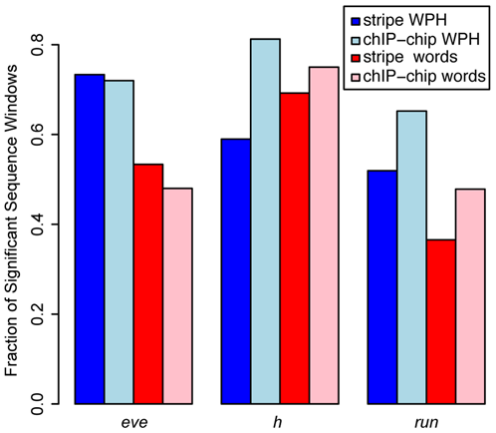
Summary of predictive power of stripe WPHs and their frequent words. For WPHs associated with stripe CRMs or with chIP-chip bound regions found near the primary pair-rule genes, most demonstrate significant overlap (*p*≤0.05) with stripe CRMs (dark blue, light blue). Words overrepresented in these WPHs also correspond well with predicted TFBSs (*p*≤0.05, red, pink).

Stripe WPHs exhibit significant overlap with these test sets, while surrounding non-coding WPHs generally do not (Figures 2–3, S1, S2). Peaks that do not correspond to the reported minimal enhancers largely correspond to 1% FDR chIP-chip bound regions (Supporting File S2), suggesting that some of these binding sites occur outside of minimal enhancers. This finding is corroborated by the modeling of sequences upstream of *eve* [14]. These results demonstrate that CRM word profiles can be used to specifically predict other CRMs under similar regulatory control.

By contrast, only portions of the *runt* stripe CRMs demonstrate predictive power (Figure S2). Since our method relies on identifying similar combinations of regulatory signals within the same short sequence window, each *runt* stripe word profile may not capture the sparse binding sites known to span these CRMs [15]. However, the additional stripe peaks found along the *runt* stripe 1+7 CRM that are absent from the chIP-chip test sets suggest that these WPHs are composed of binding sites for transcription factors other than the four considered. This observation highlights another feature of our method: the binding motifs of a given CRM need not be explicitly known to identify co-regulated sequences since the entire sequence is used.

**Figure 4:**
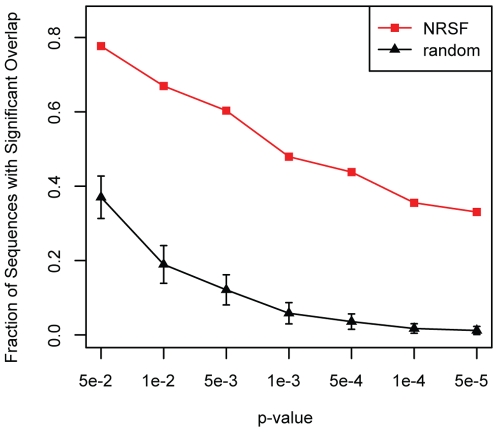
Significance of overlap between NRSF WPHs and test sets. WPHs are collected for windows spanning NRSF-bound sequences. At all *p*-value cutoffs considered, these NRSF WPHs significantly overlap with the NRSF dataset considerably more than they overlap with randomly generated test sets.

Our second dataset is drawn from the NRSF-bound sequences found on chromosome 19 in the human genome specified by the analysis of chIP-seq binding data (see *Methods*, Supporting File S3). For each 500 bp window (shifted by 100 bp) spanning these sequences, we calculated the significance of overlap between its corresponding WPH set and the NRSF-bound sequences. To ensure the degree of overlap we observed was not due to noisy pairwise matches, we repeated this calculation between each WPH set and 100 randomly generated test sets. NRSF WPHs are far more predictive of other NRSF sequences than of our random test sets (Figure 4). However, the degree of false positives at higher *p*-values indicates that there are a large number of noisy pairwise hits across chromosome 19.

### Shared words among stripe WPHs correspond to known TFBSs

Each set of WPHs consists of sequences that are similar in word composition to a single seed sequence. To determine whether WPHs share words that correspond to known regulatory sequence signals, for each set of WPHs, we collected the words in the seed sequence whose 1-neighbors are most commonly found among the WPHs and compared these words to predicted TFBSs (see *Methods*, Supporting File S4). These frequent WPH words often correspond to TFBSs predicted across the stripe CRMs (Figure 3). However, only 17.8% of the NRSF-bound sequences containing an NRSE (the sequence bound by NRSF) have a window with significant (p≤0.05) overlap between its NRSE its frequent WPH words. This percentage increases to 68.6% when only considering frequent words among NRSF hits in each WPH set, which suggests that the false positives in these WPH sets are likely masking NRSE signals. These results demonstrate that the false positives in larger genomes are a significant hurdle to isolating relevant regulatory sequence signals from WPHs.

### Word profiles of stripe CRMs recover orthologous CRMs in distant species

To study the subtleties of sequence evolution, sequencing projects are currently underway to sequence closely related genomes. These studies require tools to translate existing annotations to the new related genomes. Regulatory sequences can be a particularly difficult sequence feature to translate, as they tend to be in more flux than coding sequences, and any organizational constraints they are subject to are not well understood. Alignments have proven unreliable for some enhancers, especially in distantly related species. The accurate identification of regulatory sequences in related species is critical to understanding their evolution as well as the intricacies of the regulatory code.

**Figure 5:**
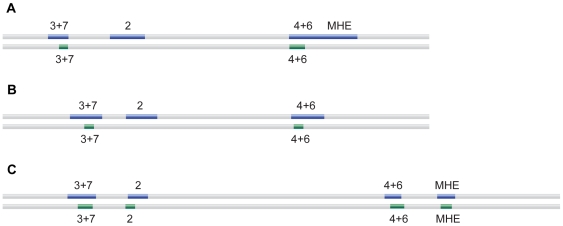
Identifying orthologous CRMs in distant fly species. We scanned *eve* CRMs from *D. melanogaster* against the eve loci of several distant fly species, *Sepsis cynipsea* (A), *Themira putris* (B), *Scaptodrosophila lebanonensis* (C). The upper blue track indicates experimentally verified CRMs, the lower green track shows the best match to the indicated *D. melanogaster* CRM. The best match is not shown if the score did meet the score threshold (*Z*≥6).

Several *eve* enhancers (stripe 3+7, stripe 2, stripe 4+6 and MHE) have been manually identified in sequences of the *eve* locus of several distantly related fly species (*S. lebanonensis*, *T. putris*, *T. superba*, *S. cynipsea*) which have minimal non-coding similarity to *D. melanogaster* [16] (Supporting File S5). Although the manual methods used to identify these enhancers were successful, they do not scale well. Using *eve* enhancers from *D. melanogaster*, we scanned the scaffolds on which *eve* is found for the tested species. All four of the *eve* enhancers were identified in *S. lebanonensis* and S. *cynipsea*, but only the stripe CRMs were verified in *T. putris*, and only the MHE CRM was verified in *T. superba*. Figure 5 summarizes our results: we recovered 8 of the 12 verified enhancers, detecting all of the CRMs for the more closely related *S. lebanonensis*, but only the stripe 3+7 and stripe 4+6 CRMs in the remaining species. These findings illustrate the ability to identify orthologous CRMs in species that have diverged approximately 120 million years ago.

### Local word profile clustering is predictive of stripe CRMs

**Figure 6:**
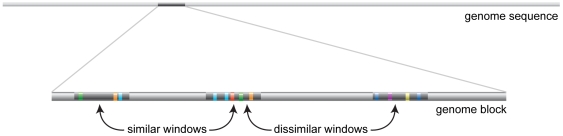
Finding similar and dissimilar sequence neighbors (HSNs and LSNs). Given a block size *B* and a threshold pairwise score of similarity, we scanned the genome for sequence windows with either high-scoring or low-scoring neighboring sequences within *B* kb.

The stripe CRMs are examples of regulatory sequences that are not only modular, but are also somewhat repetitive: these CRMs are regulated by a similar set of transcription factors and are found clustered together in the genome. Although many regulatory sequences are known not to operate under these design principles, we attempted to recover CRMs that are by searching for local word profile clustering. This search is based on our pairwise similarity score: a genome window is considered a putative clustered CRM if it has a high pairwise score with another nearby non-overlapping window within *B* kb (see *Methods*, Figure 6).

**Figure 7:**
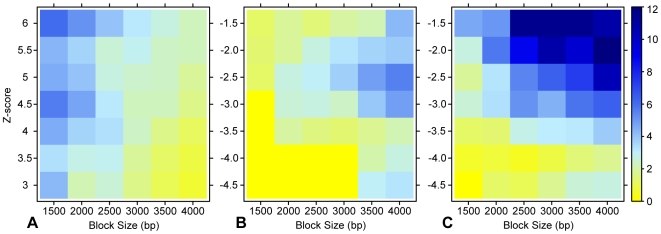
Significance of overlap between HSNs/LSNs and REDfly CRMs. Each set of HSNs (or LSNs) represents sequences with a high-scoring (or low-scoring) neighbor within a given block size for a given *Z*-score threshold. The significance of overlap between an HSN (or LSN) set and known CRMs is represented by a color scale (−log(*p*)), such that blue shades represent significant enrichment of CRMs (*p*<0.05). While HSNs are enriched for stripe CRMs (A), they are not enriched for the broader set of CRMs in REDfly (*p*>0.05 for all block sizes and *Z*-scores, data not shown), suggesting that CRM clustering is not a common feature of CRM organization. LSNs are enriched for both stripe CRMs (B) and REDfly CRMs (C) at modest score cutoffs. For *p*<1e-5, the *p*-value is reported as 6.1e-6 (−log(*p*) = 12).

Using several score cutoffs and neighborhood (*B*) sizes, we collected sets of high-scoring neighbors (HSNs), or sequences with a high-scoring neighbor within a given neighborhood size. We calculated the significance of overlap between these HSN sets and two CRM test sets, the REDfly CRMs (Supplementary File S6) and the well-studied stripe subset of REDfly (Supplementary File S1). Although HSNs for smaller block sizes are enriched for stripe CRMs (Figure 7A), they are not enriched for REDfly CRMs (*p*>0.05 for all block sizes and *Z*-scores, data not shown), suggesting that the repetitive modular regulatory sequences characteristic of stripe CRMs are not a common design feature of many CRMs.

### Locally dissimilar sequences are predictive of REDfly CRMs

**Figure 8:**
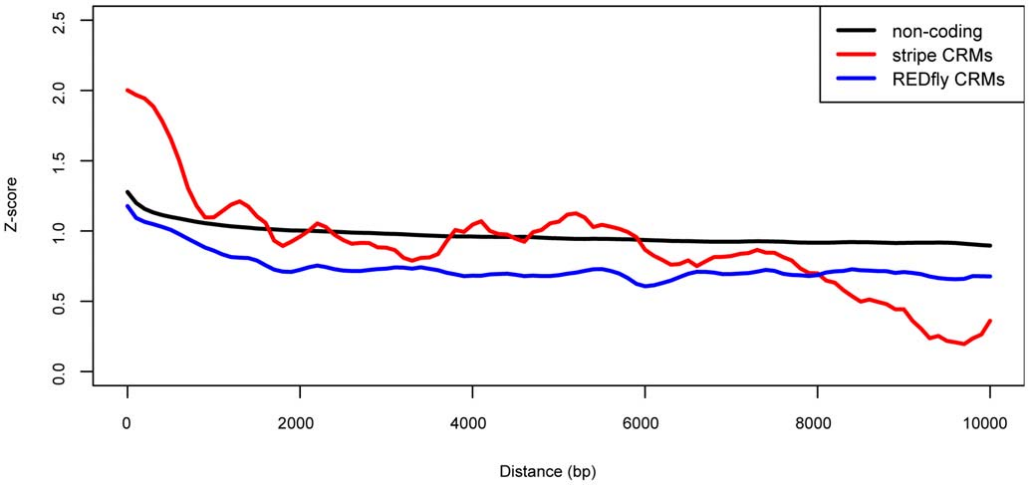
Average pairwise Z-score as a function of distance. Pairwise sequence similarity decreases as the distance between the two sequences in the genome increases. On average, non-coding sequences (black) are more similar to neighboring sequences than REDfly CRM sequences (blue). Stripe CRMs (red), known to cluster together, are similar to close neighboring sequences.

We looked more closely at the neighborhood of CRMs as measured by our similarity score to see if the level of local similarity near REDfly CRMs is indistinguishable from that of non-coding sequences. As suggested by our findings with sets of HSNs, stripe CRMs have high-scoring neighboring sequences on average relative to non-coding sequences (Figure 8). Non-coding sequences also appear to be more similar to its neighbors than expected by chance based on our scoring scheme. This finding may illustrate the degree to which non-coding sequences in *Drosophila* are inherently repetitive, beyond what is annotated by Flybase and RepeatMasker. More precisely, it is likely that the non-uniform distribution of microsatellites in the *D. melanogaster* genome contributes to the observed level of local similarity, as we do not explicitly correct for biases in dinucleotide and trinucleotide frequencies [17]. By contrast, REDfly CRMs have relatively low-scoring neighbors. This observation may be due to measurable differences between regulatory sequences and their flanking non-regulatory sequences or other nearby CRMs that do not share similar binding sites.

We used this feature of REDfly CRMs as a criterion for identifying regulatory sequences *de novo*. Instead of looking for sets of high-scoring neighbors, we scanned the genome for sequences with low-scoring neighbors (LSNs, Figure 7B–C). At modest score cutoffs and broad neighborhood sizes, LSNs are strongly enriched for REDfly CRMs. LSNs are also enriched for stripe CRMs for larger block sizes.

**Figure 9:**
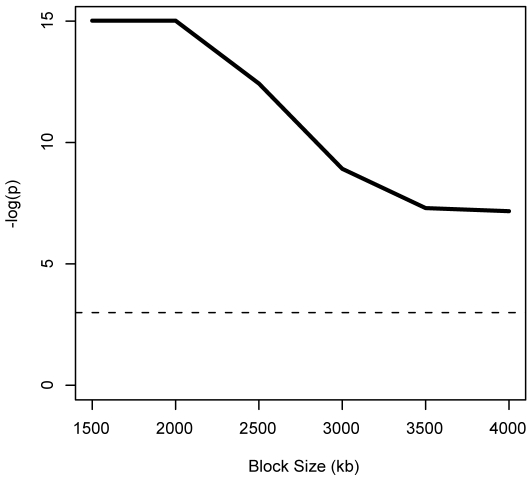
Significance of overlap between low-scoring neighbors of REDfly sequences and REDfly CRMs. Low-scoring (*Z*≤−1.5) neighbors of REDfly CRMs overlap REDfly CRMs more than expected by chance compared to its coverage of “valid” non-coding sequences surrounding REDfly CRMs. For *p*<5e-7, the *p*-value is reported as 3e-7 (−log(*p*) = 15). The dashed line represents *p* = 0.05.

To determine whether these CRMs are dissimilar to other nearby regulatory sequences, we looked for enrichment of REDfly CRMs in sequences that are dissimilar neighbors (*Z*≤−1.5) of REDfly CRM sequences. The overlap of these neighbors with REDfly CRM sequences is significant (*p*<0.001) for all block sizes considered (Figure 9). Larger block sizes may include more distant CRMs that are not identified as CRMs in the REDfly database. This finding suggests that differences between nearby regulatory sequences may account for the decreased similarity among sequences surrounding REDfly CRMs versus non-coding sequences on the whole (Figure 8).

## Discussion

We have presented WPH-finder, a means of looking for co-regulated sequences given a known CRM in the absence of explicit TFBS models. Given a known CRM, a genome-wide scan of its word profile returns a small handful of sequences (typically<1% of non-coding sequences) that are found to significantly overlap CRMs known to be under similar regulatory control. The vast majority of existing methods rely on prior knowledge about the binding profiles of particular transcription factors, conservation information, or other known co-expressed sequences, information which may or may not be available. Despite success in leveraging binding site clustering as a predictive measure of regulatory potential, either by looking for overrepresentation of known or derived motifs [2]–[3], [18], it is not clear that this is the only means of organizing regulatory signals. [7], [19]. Similarly, although conserved sequences have been shown to correspond to true regulatory sequences, there are other known regulatory sequences that are not well-conserved, even within functional binding sites, or whose conservation levels are similar to those of other non-coding sequences [20]–[21].

In light of these findings, it is a tantalizing problem to identify other sequence signals to help us identify novel CRMs that do not rely on these fallible sources. Other *de novo* approaches make use of word frequencies to look for overrepresentation of word motifs [8]–[10], or use comparative data to look for conserved CRM subsequences [22]–[24]. We use a simple word-based motif model and a straightforward score of pairwise sequence similarity as an alignment-free and efficient means to look for similar regulatory sequences, without relying on orthologous sequences or an explicit requirement of motif overrepresentation. Such a model greatly simplifies the degeneracy of binding motifs, and therefore we expect to miss CRMs whose motifs exhibit low information content. A position weight matrix (PWM) offers a much more precise description of the binding preferences for a given transcription factor, but the total space of all possible PWMs across each subsequence of a genome cannot be completely explored in an efficient manner. Despite using a limited motif model, our genome-wide word profile scans are able to recover known co-regulated CRMs. Furthermore, these recovered sequences share word motifs that correspond to predicted TFBSs of the original CRM.

We note that our pairwise score is similar to the D2z score applied to comparing regulatory sequences [25]. Unlike the D2z score, our score corrects for GC-skews without an explicit Markov chain background model and makes the simplifying assumption of word independence, which allows for its efficient calculation in genome-wide scans while allowing for mismatches. Our score also avoids overcounting repetitive sequences by allowing each *k*-mer to contribute only once to the similarity score and permits mismatches.

A strength and weakness of our method is using only one CRM sequence to search for co-regulated CRMs. A CRM can be isolated without prior knowledge of its regulators or which genes are co-expressed with its target genes, such as those CRMs identified by deep conservation with greatly diverged species [26]–[28]. Without such prior knowledge, sequences that are co-regulated with this CRM cannot be identified with other existing computational methods.

While we do not require a set of co-expressed sequences to produce meaningful hits, our method is subject to noisy pairwise matches and is unlikely to capture all sequences that share a common set of input TFBSs. Another source of false positives can come from recent duplications in the genome, whereby paralogous genes may fall under different regulatory controls but share similar word motifs due to lack of evolutionary distance. Since our method for finding co-regulated sequences is predicated on using the word profile of a known seed regulatory sequence, we should pick up paralogous regulatory sequences in the same WPH set only if those sequences share more word motifs with the seed sequence than expected by chance. Thus, the co-occurrence of these paralogous regulatory sequences in the same WPH set is evidence that these sequences may still be under similar regulatory controls.

Our method is unique in that we can mine the genome sequences similar to a single CRM sequence to uncover shared words. These words are good candidates for TFBSs, which in turn can be used to filter out noisy sequence hits. Thus, despite starting out with a single CRM, our method can overcome some of its limitations via post-processing and analysis of genome-wide hits. These analyses may also prove useful in identifying transcription factors in otherwise uncharacterized CRMs, such as those uncovered by deep conservation. Meaningful hits can also be filtered from those recovered across the genome by looking at non-coding sequences surrounding known co-regulated genes, which may be especially useful to reduce false positives hits in large genomes. We do not explicitly enforce this constraint, as this data is not always readily available or reliable.

We also showed that our model could be used towards identifying orthologous hits in greatly diverged sequences. The flexibility afforded regulatory sequences, believed to be critical for diversity and evolutionary change, can yield unreliable alignments for large evolutionary distances between CRMs. Searching for similar word profiles in distant species provides an alignment-free and highly specific means of looking for conserved motif combinations that may have been greatly permuted.

In an attempt to exploit local CRM clustering, we looked for novel CRMs by looking for local word profile clustering. Although we recovered many stripe CRMs, we failed to recover the vast majority of REDfly CRMs. This result suggests that clustering of CRMs may be a feature of stripe CRMs, but not one of CRMs in general.

Interestingly, our more successful CRM screen utilized the opposite approach, looking for dissimilar neighboring sequences. REDfly CRMs are measurably dissimilar from its neighboring sequences, and oftentimes, their dissimilar neighbors correspond to other REDfly CRMs. These findings suggest that these nearby sequence dissimilarities may be a universal feature of CRM organization, as evolution may favor differentiating different CRMs from each other, especially those located near each other. This feature may be even more critical in compact genomes such as *D. melanogaster*, where much of the genome appears to be under functional constraint and it is therefore more likely for functional elements to be located near each other [18].

The stripe CRMs are well-characterized and share spatial and temporal expression in embryonic development, making them an extremely useful test set in identifying common TFBSs and common properties of regulatory sequences. However, the disparity in these two approaches underscores the need for insight in other regulatory systems to learn more general characteristics of CRMs and their organizational properties. Further experimental investigation will allow computationalists to train more robust CRM-finding algorithms, which in turn will provide greater insight into the evolution of regulatory sequences.

## Methods

### Pairwise similarity score

A word profile of a sequence is defined as its 8-mer composition. Each 8-mer in the sequence is considered equally, except to correct for base composition skews as described below. The similarity between two sequences is determined by comparing the degree of word overlap between two profiles with the expected overlap given the number of words in each sequence. More precisely, for two sequences *A* and *B*, a word *w* of length *k* (*k* = *8*) in *A* contributes to the observed word overlap *ov_A→B_* if a 1-neighbor of *w* occurs in *j*. A 1-neighbor of *w*, *w′*, is defined as a word that has no more than one mismatch with *w*. Note that each pair of sequences defines two overlaps (i.e., *ov_A→B_* and *ov_B→A_*), and accordingly, there are two resulting scores, z*_A→B_* and *z_B→A_*. The overall pairwise similarity between two sequences is defined as the minimum of these two scores. Taking the minimum ensures that similarity requires many words in *A* to have 1-neighbors in *B* and vice versa. For simplicity, we proceed by showing the derivation of overlap score z*_A→B_*, which is clearly equivalent to the derivation of *z_B→A_*.

We used the Poisson distribution to calculate the probability of these overlaps. Let *W(A)* be the set of all words found in sequence *A*, and *W′(A)* be the total set of words in the 1-neighborhood of *W(A)*, including duplicates. Given *n* unique words for fixed word length *k* (i.e. *n* = 32,896 for *k = 8*; a word maps to itself and to its reverse complement), the probability that a given word *w* occurs at least once in *A* is
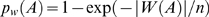



Similarly, the probability that a 1-neighbor of a given word *w* occurs in *A* is
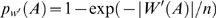



The probability that a given word *w* occurs in *A* and has a 1-neighbor in *B* is then calculated as
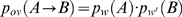



Let 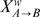 be the indicator variable that represents whether *w* occurs in *A* and *w′*, a 1-neighbor of *w*, occurs in *B.* We make the simplifying assumption that each word occurs independently, which suggests a binomial distribution with the following characteristics:
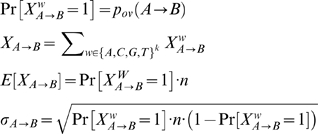



The pairwise overlap score *z_A→B_* is then defined as the corresponding z-score, which compares the degree of actual overlap, *V_A→B_*, to that of what is expected, *E*[*X_A→B_*]. Pairs of sequences with significant overlap will have high positive scores, while the expected z-score of between a pair of unrelated sequences is zero.
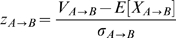



As stated above, the overall pairwise similarity score *Z(i,j)* is then calculated as
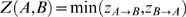



Accounting for composition bias

One of the major complications we encountered upon applying this scoring scheme is that sequences with similar GC-content would preferentially cluster together. This problem arises because the above scheme assumes that each word is equiprobable. However, sequences with significant skews in base composition will have skewed word occurrence probabilities. We corrected for GC-biases by binning together words with equal GC-ratios and calculating the probability of word overlap for each bin. This correction allows sequences with similar GC-content to have a higher probability of overlap, thereby reducing the observed GC-biases.

For a fixed word length *k*, there are *n_r_* words for each GC-ratio *r* = 0, 1/*k*, 2/*k*, …, 1. Let *W_r_(A)* be the set of words in *A* with a GC-ratio of *r*, and *W'_r_(A)* be the set of words in the 1-neighborhood of *W_r_(A)*. The word occurrence probabilities for a given GC-ratio *r* is
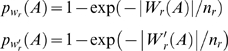



The corresponding pairwise word overlap probability between sequences *i* and *j* for words with a given GC-ratio *r* is
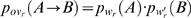



The overall probability of word overlap sums over all possible GC-ratios:
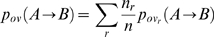



The overlap *Z* score is calculated as before, based on this overlap probability.

The *D. melanogaster* genome is highly AT-rich, with many runs of A's occurring in the non-coding regions. To avoid overcounting words associated with this ubiquitous sequence, we remove all words within the 1-neighborhood of AAAAAAAA from consideration. The word counts (*n_r_* and *n*) reflect this omission (i.e. *n* = 32,871, *n_0_* = 127, *n_1/8_* = 1008 for *k* = 8). As an additional safeguard against stretches of repetitive sequences and microsatellites, if the same occurrence of a given word *w* in sequence *A* overlaps with a previous occurrence, only the first occurrence counts towards *W(A)*. For example, for long tandem repeats of the microsatellite CAA, only the first occurrence of overlapping 8-mers (i.e. CAACAACA, AACAACAA, and ACAACAAC) is counted towards the total count of words.

To ensure that these measures correct for base composition biases, we compare pairwise scores (i.e., *z_A→B_*, *z_B→A_*) with and without taking GC skews into account over several sets of random sequences. Each of the 1000 sequences in a given random set is generated by a 0th order Markov model based on a GC content chosen from a normal distribution. The first random set has a distribution of GC ratios based on the observed distribution of random *D. melanogaster* 500 bp sequences (*μ* = 0.41, *σ^2^* = 0.06). The base composition of the second set is unskewed, representing sequences whose scores do not benefit from these base composition correction measures (*μ* = 0.5, *σ^2^* = 0.03). The last set represents the reciprocal of the first set, where sequences are GC-rich (*μ* = 0.59, *σ^2^* = 0.06). Figure S3 illustrates that our new scoring scheme indeed corrects for skews in base composition.

The sequence composition skews we encountered upon applying our scoring scheme to human sequences exceeded those found in fly sequences, with background word distribution skews not easily accounted for by base composition or by overlapping repeats alone. To more accurately compute pairwise scores between human sequences, we allow for the input of arbitrary background word frequencies. Words are binned on both frequency and GC content, such that the standard deviation of word frequencies in a given bin does not exceed a tenth of the mean and large frequency bins (>2000 words) are subdivided by GC content. Bins closely related in GC content and frequency are merged to ensure that each bin has at least 20 words. Background word frequencies of chromosome 19 are obtained by scanning non-repetitive sequences. Overlapping copies of the same word are not counted, and the final background frequency of a given word is set as the average of the frequencies of its 1-neighbors. Probabilities are calculated as described above, such that the probability of overlap is determined for each bin and weighted accordingly in the overall probability calculation. The WPH-finder program is available as Supporting File S7, as well as updated on the web (http://rana.lbl.gov).

### Sequences

Our analysis was performed on the *D. melanogaster* genome Release 4.3 (http://flybase.net). We masked the genome for CDS, repeat regions, transposons, rRNA, and tRNA as annotated by FlyBase, and for repeat regions as reported by the UCSC Genome Browser RepeatMasker track (http://genome.ucsc.edu). All of our analyses make predictions on 500 bp sequence windows shifted by 100 bp across the masked genome. Since much of the genome is masked, only “valid” windows, or windows containing at least 300 unmasked words (60% of the window size), were considered in generating prediction sets. “Valid” sequences cover 92.2 Mb of the *D. melanogaster* genome.

Our CRM datasets are the REDfly database (Supporting File S6) and the stripe CRM subset found within REDfly (Supporting File S1) for the primary pair rule genes, *eve*, *h*, and *run* (http://redfly.ccr.buffalo.edu, accessed April 2007). Overlapping CRMs were consolidated, and we excluded those that were longer than 3 kb or shorter than 300 bp. Some CRMs were heavily masked for coding and repetitive sequences (as described above). Unmasked sequences that are less than 100 bp and are found between masked sequences are masked, and masked sequences that are greater than 200 bp are removed. The resulting REDfly dataset covers 229.6 kb of the genome over 196 consolidated CRMs. The stripe subset consists of 11 consolidated CRMs covering 13.5 kb.

The chIP-chip binding datasets are drawn from [12] for transcription factors Bcd, Gt, Hb, and Kr (Supporting File S2). Each of these four datasets consists of the 500 bp windows surrounding the 1% FDR peaks near the nine pair rule genes (*eve*, *ftz*, *h*, *odd*, *opa*, *odd*, *prd*, *run*, *slp1*, *slp2*). ChIP-Chip peaks for each of the four transcription factors above are found near all of the 9 pair-rule genes and cover 10.5–21.5 kb of the genome. As in the REDfly sets, long stretches of masked sequences were excluded.

Our human data set consists of the NRSF-bound chIP-seq sequences found on chromosome 19 as reported by Wold et al [29]. Chromosome 19 has the highest concentration of NRSF-bound sequences. Long (≥200 bp) and trailing sequences that are annotated as repetitive by the UCSC genome browser (release hg17) are removed from the set. After removing repetitive sequences, sequences shorter than 300 bp are removed, as we require at least 60% of the words in a 500 bp window to be unmasked. Our final dataset consists of 121 unique sequences spanning 82,829 bp, 118 of which contain an annotated binding site, NRSE (Supporting File S3).

### Finding co-regulated CRMs (WPHs)

A set of WPHs is defined as a set of sequences that are all pairwise similar to a given seed sequence by our pairwise similarity score described above (Figure 1). In our analysis of stripe CRMs, we used a similarity score threshold of *Z*≥5, and seed sequences are 500 bp sequence windows across the *eve*, *h*, and *run* regulatory regions shifted by 100 bp. These regulatory regions were chosen to encompass 15 kb surrounding known stripe CRMs (*D. melanogaster* release 4.3 coordinates: *eve*, chr2R:5,485,827-5,500,826; *h*, chr3L:8,634,112-8,649,111; *run*, chrX:20,487,522-20,502,521). These regulatory regions and the WPHs are drawn from the masked genome described above.

We extended the 121 NRSF-bound sequences such that each sequence in the dataset is a multiple of 100 and the minimum length is 500 bp. As with the stripe CRMs, seed sequences are 500 bp sequence windows shifted by 100 bp across this length-extended set and are masked for repetitive sequences. We used a score cut-off of *Z*≥2 to identify WPH sets for each sequence window, and compare the overlap of each WPH set with the original NRSF dataset described in the previous section as well as with 100 randomly generated test sets. These random test sets are drawn from chromosome 19 such that each set contains 121 sequences that are length-matched to the NRSF set and have few (<10%) repetitive sequences.

### Finding shared words in WPHs

To identify shared words across WPHs, we looked at the most frequent words found among the sequences in a WPH set that are in the 1-neighborhood of the words in the original seed sequence. Given the background genome word frequency for each word *w* in the seed sequence, *f(w)*, we normalized *f(w)* with respect to all words in the seed sequence, *f'(w)* = *f(w)/*∑*_w_ f(w),* and we scored each word based on the difference between the expected frequency of *w* and its observed frequency in the WPH set, log (*f'(w)/f_obs_(w)*). Each word is also assigned a score based on its frequency in repeat sequences, *f_r_(w)*, allowing words that look repetitive, log (*f(w)/f_r_(w)*)<0.2, to be removed from consideration. The top *m* words are reported as the set of overrepresented words in a WPH set. Since the median coverage of an NRSE is 21 bp while the stripe CRMs are known to be densely populated with binding sites, we set *m* = 5 for the stripe WPHs and *m* = 3 for the NRSF WPHs.

We looked at the overlap of these words with predicted binding sites of transcription factors known to be involved in early stripe patterning. These TFBSs were identified across the stripe CRMs with Patser [16], using empirically determined score cutoffs (Bcd -6; Hb -6; Gt -5.5; Kr -6; Slp1 -6; Kni -6.5; Dstat -6; see Supporting File S4). The predictions of NRSF binding sites (NRSE) are taken from [29].

### Finding orthologous CRMs

Scaffolds of distant fly species (*Scaptodrosophila lebanonensis*, *Themira putris*, *Themira superba*, *Sepsis cynipsea*) were made available through a sequencing project with the Joint Genome Institute [16]. The genomic locations of some of the *eve* enhancers have been verified by transgenic experiments and were manually identified by careful inspection of dotplots with orthologous *D. melanogaster* sequences [16] (Supporting File S5). To identify orthologous CRMs using their word profiles, the entire scaffold on which the target gene is found was scanned against the desired query CRM from *D. melanogaster*, in the same manner that we look for WPHs (see above). The 500 bp window with the best match to each 500 bp CRM window was returned, provided that the best match exceeds a high threshold (*Z*≥6).

### Finding locally similar and dissimilar sequences (HSNs and LSNs)

Sequence neighbors are defined as non-overlapping sequence windows that are found within the same sequence block, or within *B* kb of each other (*B* = 1.5, 2, …, 4). For a given block size *B* and score cutoff *Z*, a set of HSNs (or LSNs) is defined as all “valid” sequence windows in the genome (see above) with a high-scoring (or low-scoring) sequence neighbor.

To determine appropriate score cutoffs, we looked at the distribution of pairwise scores between 1000 “valid” sequence windows (Figure S4). These sequence windows are randomly chosen such that each pair of windows is separated by at least 50 kb to avoid comparing neighboring sequences. To capture sequences that occur ∼5% by random, we used score thresholds of *Z*≥3 to collect sets of HSNs and score thresholds of *Z*≤−1.5 to form sets of LSNs.

### Assessing predictive power

Given a set of sequences that are putative regulatory sequences (i.e., WPHs, HSNs, LSNs), we evaluated their predictive potential by computing the significance of their overlap with one or more test sets. We calculated *p*-values by comparing this overlap with the overlap between a given test set and random sequence sets (*n* = 100,000). These random sequence sets are created by permuting the lengths and distances of sequences found in the original sequence set across the “valid” non-coding genomic sequences. This method was similarly applied to assessing the significance of the overlap between sequences that are dissimilar neighbors of REDfly CRMs and the REDfly set, aside from using only “valid” sequences that are within neighborhood boundaries of REDfly CRMs instead of all “valid” non-coding sequences across the genome (*n* = 2,000,000).

We calculated analogous *p*-values when comparing the overlap of common word signals in a WPH set with the predicted TFBSs across the seed sequence window. In this case, the seed sequence is fragmented into (overlapping) common words and the intervening spacers. Random word sets were formed by permuting the lengths of the fragments separated by spacers of random length (*n* = 100,000).

## Supporting Information

Figure S1 Significance of overlap between h WPHs and test sets. WPHs corresponding to h stripe CRM sequences significantly overlap both other stripe CRMs and chIP-chip peaks near pair-rule genes. Stripe CRMs are shaded in gray, and chIP-chip bounds regions are boxed in a dotted line. For p<1e-5, the p-value is reported as 6.1e-6 (−log(p) = 12). The dashed line represents p = 0.05.
(0.38 MB TIF)

Figure S2 Significance of overlap between run WPHs and test sets. run stripe CRM WPHs tend to significantly overlap other stripe CRMs, and chIP-chip peaks. For p<1e-5, the p-value is reported as 6.1e-6 (−log(p) = 12). Stripe CRMs are shaded in gray, and chIP-chip bounds regions are boxed in a dotted line. The dashed line represents p = 0.05.
(0.36 MB TIF)

Figure S3 GC correction eliminates GC skews in pairwise similarity scores. We generated 500 bp random sequences whose GC content is drawn from a normal distribution, and compared the distribution of their pairwise similarity scores with and without GC correction. The mean GC content of a pair of sequences is plotted against the mean Z-score for all pairs of sequences with the same mean GC content to illustrate score variance with respect to GC ratio. For sequences mimicking the GC content of the D. melanogaster genome ((A) μ = 0.41, σ2 = 0.06) and those with the reciprocal GC ratio distribution ((C) μ = 0.59, σ2 = 0.06), the uncorrected pairwise scores vary with GC ratio while the GC-corrected scores do not. Random sequences with an unskewed base composition ((B) μ = 0.5, σ2 = 0.03) do not benefit from these base composition correction measures.
(0.09 MB TIF)

Figure S4 Distribution of pairwise similarity scores. Using 500 bp windows drawn from the D. melanogaster non-coding genome, we use the histogram of the all-by-all pairwise scores to determine extreme score cutoffs. The mean and median of this distribution are 0.67 and 0.63 respectively. We suspect that the non-zero mean of these scores is due to the non-random composition of the non-coding sequences.
(0.07 MB TIF)

File S1 Stripe CRMs from REDfly. Fasta file of REDfly CRMs associated with stripe formation regulating primary pair-rule genes: even-skipped, hairy, and runt.
(0.01 MB TXT)

File S2 ChIP-Chip peaks surrounding pair-rule genes. Positions of 1% FDR chIP-chip peaks found upstream and downstream of pair-rule genes (eve, ftz, h, odd, opa, odd, prd, run, slp1, slp2) for transcription factors Bcd, Gt, Hb, and Kr. Sequence positions are with respect to release 4.3 of the Drosophila melanogaster genome.
(0.00 MB TXT)

File S3 NRSF-bound chIP-seq sequences. Fasta file of chromosome 19 NRSF-bound chIP-seq sequences used in this study, such that long repetitive sequences are removed and each sequence exceeds 300 bp.
(0.09 MB TXT)

File S4 Patser motif hits near stripe CRMs. Patser motif hits for twi, Pnt, pan, med, mad, Tin, bcd, hb, gt, Kr, slp1, kni, and Dstat across even-skipped, hairy, and runt loci.
(0.03 MB TXT)

File S5 Eve scaffolds of distant fly species. Fasta file of eve loci in Scaptodrosophila lebanonesis, Sepsis cynipsea, Themira putris, Themira superba.
(0.16 MB TXT)

File S6 REDfly CRMs. Fasta file of REDfly CRMs used in this study. Overlapping CRMs are merged, and masked and long (>3 kb) sequences are removed.
(0.24 MB TXT)

File S7 WPH-finder. Code to scan genome sequences for WPHs. Please see http://rana.lbl.gov for most current version.
(5.54 MB GZ)
