# Supplementary figures and images for: Identifying Cis-Regulatory Sequences by Word Profile Similarity
Source: PLoS One. 2009 Sep 4;4(9):e6901. doi: 10.1371/journal.pone.0006901 (PMC2731932; doi:10.1371/journal.pone.0006901)

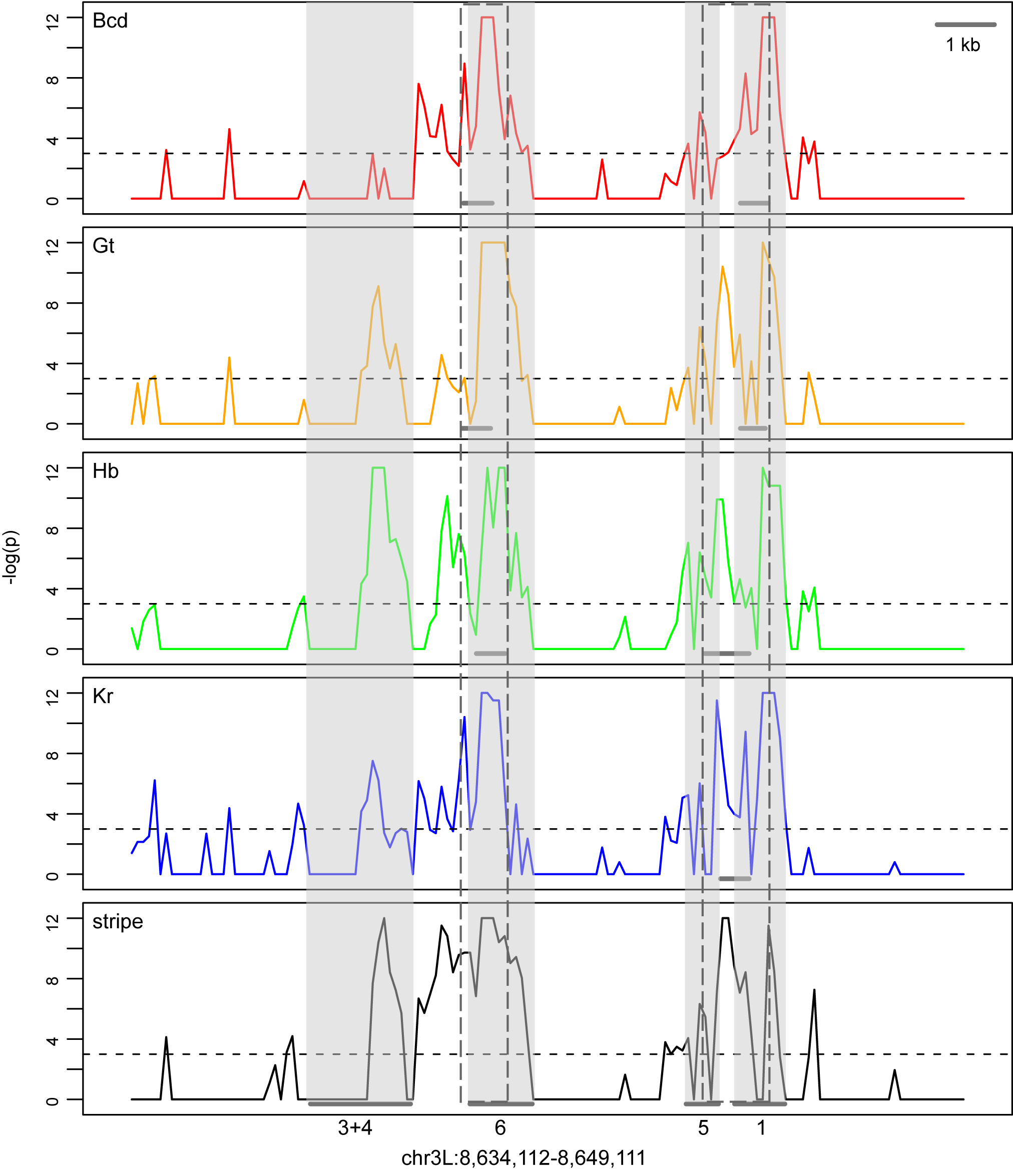

Supplement: Figure S1 — Significance of overlap between h WPHs and test sets. WPHs corresponding to h stripe CRM sequences significantly overlap both other stripe CRMs and chIP-chip peaks near pair-rule genes. Stripe CRMs are shaded in gray, and chIP-chip bounds regions are boxed in a dotted line. For p<1e-5, the p-value is reported as 6.1e-6 (−log(p) = 12). The dashed line represents p = 0.05. (0.38 MB TIF) [file pone.0006901.s001.tif]

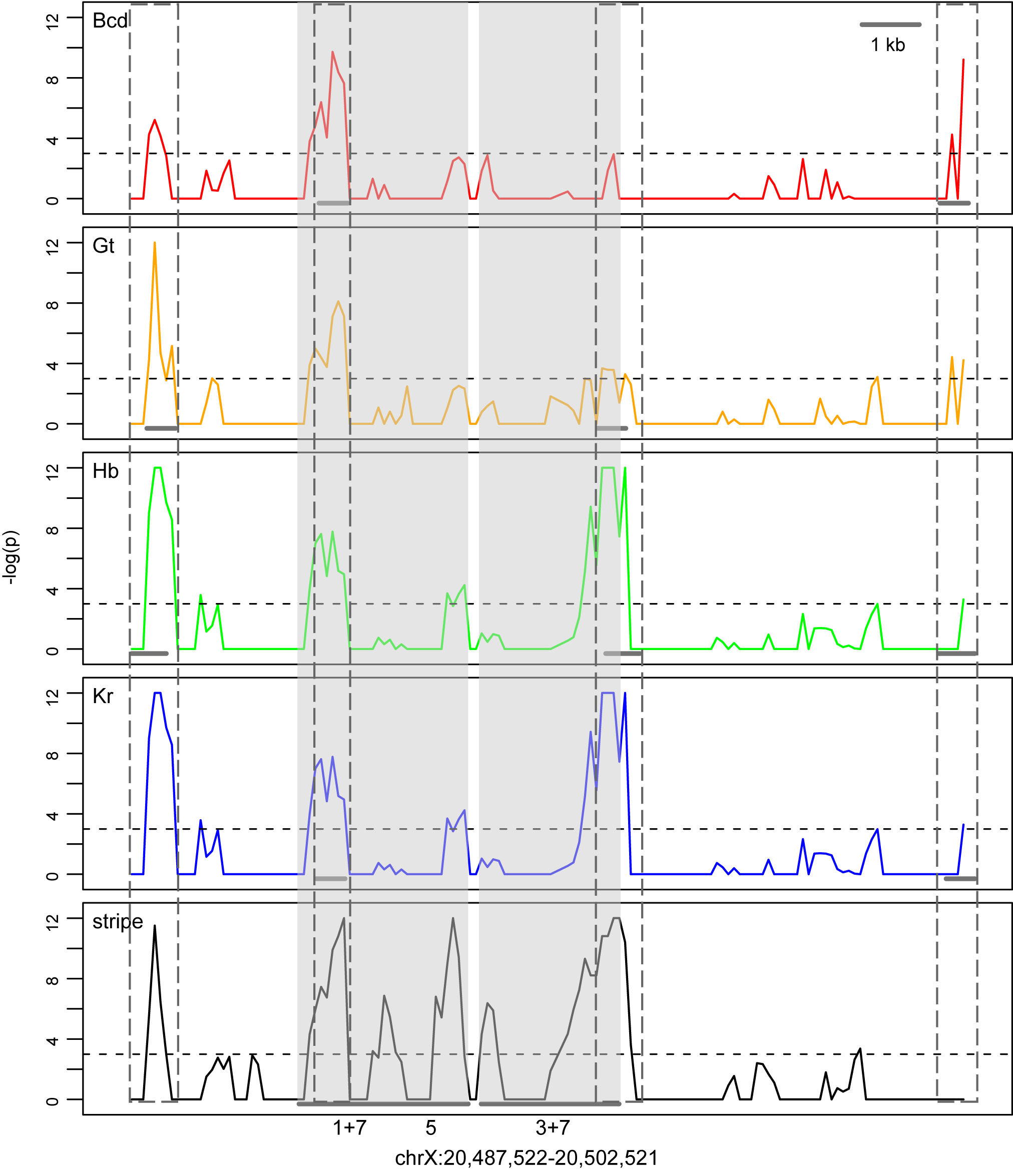

Supplement: Figure S2 — Significance of overlap between run WPHs and test sets. run stripe CRM WPHs tend to significantly overlap other stripe CRMs, and chIP-chip peaks. For p<1e-5, the p-value is reported as 6.1e-6 (−log(p) = 12). Stripe CRMs are shaded in gray, and chIP-chip bounds regions are boxed in a dotted line. The dashed line represents p = 0.05. (0.36 MB TIF) [file pone.0006901.s002.tif]

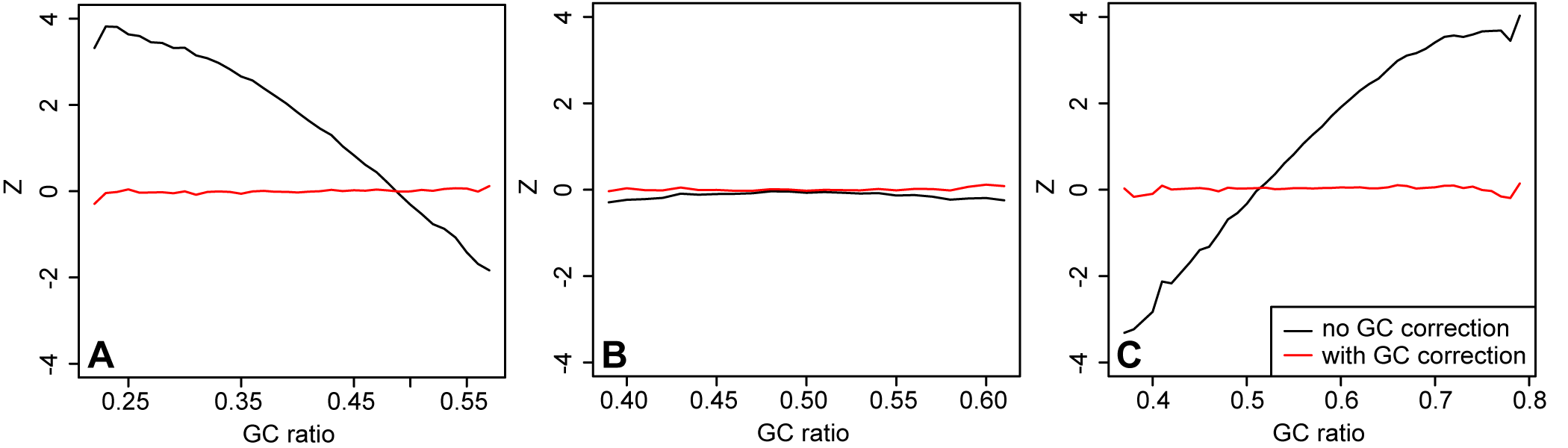

Supplement: Figure S3 — GC correction eliminates GC skews in pairwise similarity scores. We generated 500 bp random sequences whose GC content is drawn from a normal distribution, and compared the distribution of their pairwise similarity scores with and without GC correction. The mean GC content of a pair of sequences is plotted against the mean Z-score for all pairs of sequences with the same mean GC content to illustrate score variance with respect to GC ratio. For sequences mimicking the GC content of the D. melanogaster genome ((A) μ = 0.41, σ2 = 0.06) and those with the reciprocal GC ratio distribution ((C) μ = 0.59, σ2 = 0.06), the uncorrected pairwise scores vary with GC ratio while the GC-corrected scores do not. Random sequences with an unskewed base composition ((B) μ = 0.5, σ2 = 0.03) do not benefit from these base composition correction measures. (0.09 MB TIF) [file pone.0006901.s003.tif]

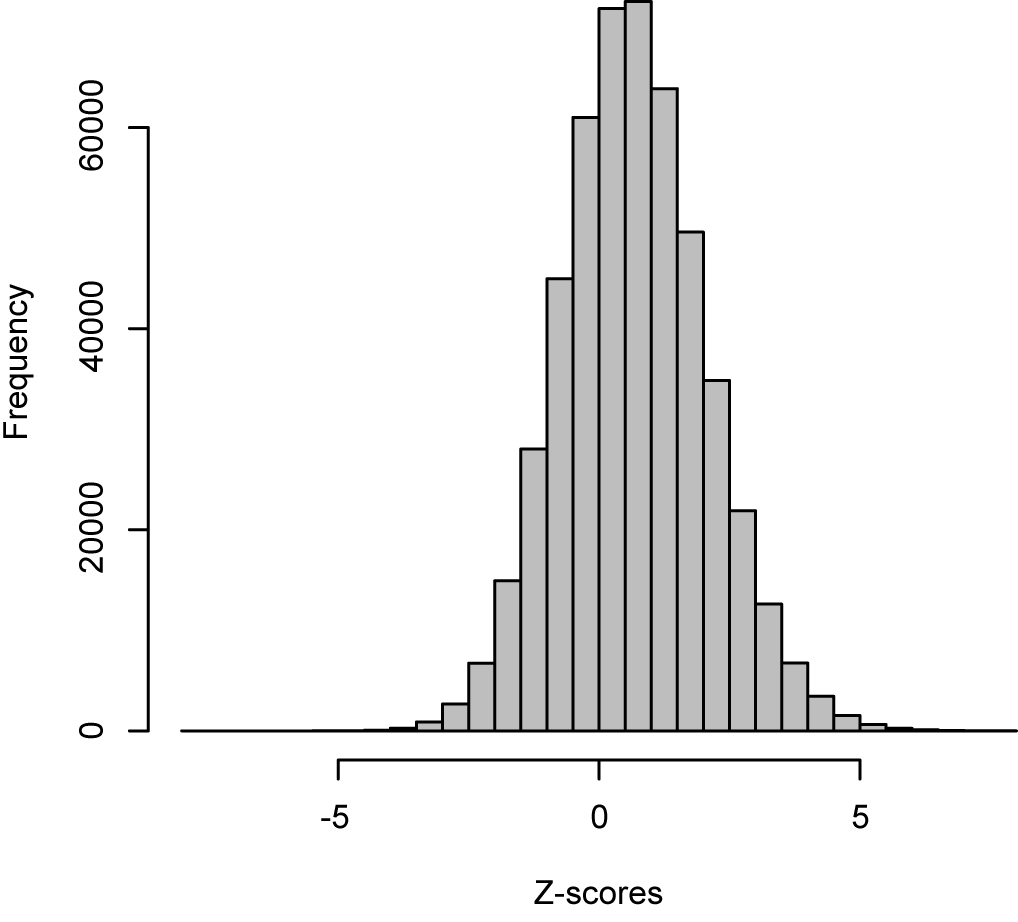

Supplement: Figure S4 — Distribution of pairwise similarity scores. Using 500 bp windows drawn from the D. melanogaster non-coding genome, we use the histogram of the all-by-all pairwise scores to determine extreme score cutoffs. The mean and median of this distribution are 0.67 and 0.63 respectively. We suspect that the non-zero mean of these scores is due to the non-random composition of the non-coding sequences. (0.07 MB TIF) [file pone.0006901.s004.tif]
